# Is the Personal Identity Debate a “Threat” to Neurosurgical Patients? A Reply to Müller et al.

**DOI:** 10.1007/s12152-017-9337-4

**Published:** 2017-06-27

**Authors:** Sven Nyholm

**Affiliations:** Eindhoven University of Technology, Eindhoven, The Netherlands

**Keywords:** Deep brain stimulation, the self, personal identity, metaphysics

## Abstract

In their article in this journal, Sabine Müller, Merlin Bittlinger, and Henrik Walter launch a sweeping attack against what they call the “personal identity debate” as it relates to patients treated with deep brain stimulation (DBS). In this critique offered by Müller et al., the personal identity debate is said to: (a) be metaphysical in a problematic way, (b) constitute a threat to patients, and (c) use “vague” and “contradictory” statements from patients and their families as direct evidence for metaphysical theories. In this response, I critically evaluate Müller et al.’s argument, with a special focus on these three just-mentioned aspects of their discussion. My conclusion is that Müller et al.’s overall argument is problematic. It overgeneralizes criticisms that may apply to some, but certainly not to all, contributions to what they call the personal identity-debate. Moreover, it rests on a problematic conception of what much of this debate is about. Nor is Müller et al.’s overall argument fair in its assessment of the methodology used by most participants in the debate. For these reasons, we should be skeptical of Müller et al.’s claim that the “personal identity debate” is a “threat to neurosurgical patients”.

In their recent article in this journal, Sabine Müller, Merlin Bittlinger, and Henrik Walter criticize what they call the “personal identity debate” as it relates to patients treated with deep brain stimulation (DBS) [1]. In this critique offered by Müller et al., the personal identity debate is said to: (a) be metaphysical in a problematic way, (b) constitute a threat to patients, and (c) use “vague” and “contradictory” statements from patients and their families as direct evidence for metaphysical theories. In what follows, I critically evaluate Müller et al.’s argument, with a special focus on these three just-mentioned aspects of their discussion. My conclusion is that Müller et al.’s overall argument is problematic. It overgeneralizes criticisms that may apply to some, but certainly not to all, contributions to what they call the personal identity debate. Moreover, it rests on a problematic conception of what much of this debate is about. Nor is Müller et al.’s overall argument fair in its assessment of the methodology used by most participants in the debate. For these reasons, we should be skeptical of Müller et al.’s general conclusion that the “personal identity debate” is a “threat to neurosurgical patients”.

The rest of my discussion proceeds as follows. First I quickly provide some background. I then introduce and critically evaluate the three different aspects of Müller et al.’s discussion identified above, finding each aspect to be problematic. I end with a brief concluding discussion.

## Background

DBS is a technology whereby surgically implanted electrodes directly stimulate specifically targeted brain areas. This targeted stimulation can modulate certain functions and behaviors. For this reason, DBS is used as a treatment for several medical conditions, including Parkinson’s disease. There are also more experimental trials underway, for example for anorexia nervosa. Across a growing range of potential uses, DBS is showing great promise.

However, the very idea of “merging” with a piece of medical technology in this way, and in effect getting an “on/off” switch between different modes of functioning, raises the question of what impact DBS might be thought to have on the self. Indeed, when interviewed about their experiences with DBS, patients and their families have voiced thoughts and concerns about apparent effects on the patient’s self. Some speak of the impact as being positive – e.g. as “a second birth” – and some as negative—e.g. “I no longer feel like myself”. In many cases, no noticeable effects on the personality or the self of the patient are observed. However, there are enough cases for neurologists and other key stakeholders to take very seriously the question of how we should interpret the relation between DBS-stimulation and a patient’s self or personal identity [2].

Accordingly, many neuroethicists have recently discussed the question of how we should interpret the effects DBS can have on the self, as well as the normative question of whether these possible effects are negative, positive, or perhaps neutral from an ethical point of view. A wide range of perspectives have been brought to bear on this general issue. Some have made use of the literature on “personal identity” with its different neo-Lockean perspectives, such as Derek Parfit’s important work about psychological continuity and connectedness over time [3]. For example, Witt et al. ask whether DBS might undermine psychological continuity over time, which they view as a potential negative side-effect of DBS [4]. Others have brought different types of “narrative” perspectives to bear on this topic. Schechtman, for example, discusses what DBS might do to a person’s self-created life-story [5]. Baylis discusses the impact it might have on a person’s public persona or “relational identity” [6]. Others, such as Kraemer and Johansson et al., have considered questions related to “authenticity” [7, 8]. Together with Elizabeth O'Neill, I have related ideas from everyday life, literature, philosophy, and social psychology about the notion of the “true self” to this issue of DBS and the self [9].

In sum, the neuroethical engagement with the effects DBS can have on a patient’s self has ranged from everything from psychological continuity over time, to a person’s life-story, to her socially constructed relational identity, to ideas about authenticity and the “true self.” It is this growing literature that Müller et al. refer to when they use the expression “the personal identity debate.” They cite all of the writers briefly mentioned above, myself included. And so we are all, I take it, intended targets of the criticism that Müller et al. launch against this personal identity debate. With this background in place, I turn now to the three aspects of Müller et al.’s discussion that I will respond to.

## Metaphysics?

One of Müller et al.’s key arguments goes like this. The contributions to this “personal identity debate” are all first interpreted as being, or being based on, “metaphysical theories.” It is claimed that metaphysical theories are “applied” to the real-life situations faced by patients and their families. It is then argued that there is wide-ranging disagreement about metaphysics and that, for this reason, metaphysical theories are not fit to be part of ethical and legal arguments within liberal societies. Therefore, it is concluded, the personal identity debate is illiberal, by being based on “contentious” metaphysics [1]. What is this supposed to mean? And is it really the case that all contributions to the personal identity debate ought to be understood as “metaphysical”?

One thing that complicates the evaluation of Müller et al.’s argument is that they never explain what they mean by “metaphysics,” even though they label all of the contributions to the personal identity debate that they review as “metaphysical.” This could mean that they use “metaphysical” as a general term of abuse, as has sometimes been done within certain types of philosophy. However, I assume that their argument has more substance than mere name-calling does. So, it must be that Müller et al. have something more distinctive in mind.

Typically, metaphysics is understood as being about the “nature of reality” on the most general level. It’s about what exists in the universe, about what could exist or happen, and it’s about the fundamental nature of entities, their properties, or events that could occur. It’s about distinguishing what is necessary from what is merely contingent. This cluster of issues is what is typically referred to under the general heading of “metaphysics” [10]. Since this is the most common understanding of what metaphysics is, I will assume that this is what Müller et al. mean when they talk about metaphysics.^1^ Is the whole personal identity debate metaphysical in this common way of understanding what metaphysics is about? I think that it is not.

Many of the contributions to the personal identity debate that Müller et al. include under the umbrella of “metaphysical theories” are not best understood as metaphysical in the just-described standard sense. They are instead better understood as being about people’s attitudes and values. Or they are about how people interpret themselves and others, or about the practical identities they create for themselves through the choices they make.

Take Schechtman’s theory of “narrative identity” as a first example. This theory is about making choices and having attitudes that help to form a distinctive “narrative” (or “life-story”) that a person sees as constituting his or her identity [5]. Or take Baylis’ variation on this theme: the idea of a person’s “relational identity.” This has to do with how people view us and how we want and try to portray ourselves both in relation to our own desires and our beliefs about how other people perceive us. A person’s relational identity, on this view, is a form of social construction where both other people and the person him or herself are authors or creators of that identity [6]. Neither of these theories – both of which Müller et al. have as part of their list of “metaphysical” theories – are most naturally interpreted as metaphysical conceptions of identity. Rather, they are about the ways in which we interpret ourselves and other people, and about the practical identities and life-stories we create for ourselves.

Consider next the idea of the “true self”, as I was discussing it in my article together with Elizabeth O'Neill. When Müller et al. mention our article, they say that we were discussing “the metaphysical term the “true self”.” In our article, however, we were arguing that a person’s idea of his or her true self depends crucially on what he or she values. That is, people tend to associate aspects of themselves they value with their “true self” and aspects of themselves they see as less valuable with more peripheral aspects of their self [9]. We argued for this using some recent work on judgments about people’s true self from experimental philosophy and social psychology, by writers like Newman et al. and Strohminger et al. [11, 12]. They too argue that people’s judgments about what constitutes somebody’s true self tend to depend importantly on their values. This is not best described as “metaphysics”. It rather has to do with people’s values.

The related idea of “authenticity”, which Müller et al. also have as an example of something metaphysical, is also better understood as a value or value-judgment, rather than as a metaphysical concept. This can be so whether we take an “essentialist” or “existentialist” view of how to interpret the value of authenticity – or perhaps some combination of the two [13]. As Neil Levy writes in a related discussion about authenticity:

We can emphasize self-discovery without holding the empirically implausible notion that the self has a fixed essence; we can point to the fact that people *do* have dispositions and talents and personalities, which fit them better for some activities than for others. .. without committing ourselves to the claim that people are immutable, and even without denying that genuinely profound change is possible. We can emphasize self-creation without denying that change is difficult and always only partial. The ethics of self-creation and of self-discovery are better seen as outlooks on human life; conceptions of how we best live [14].

Pugh et al. argue in forthcoming work that we can directly import what Levy says above into the context of the personal identity debate regarding DBS [13]. In other words, we can interpret people’s thoughts about self-creation and self-discovery as these are related to DBS as expressing outlooks on human life, or conceptions of how best to live. None of this is best interpreted as metaphysics. It is rather a matter of values and ethics.

## A Threat to Patients?

Another key argument in Müller et al.’s article goes like this. It is first claimed that some theories of personal identity can be used to argue against honoring advance directives under some circumstances. The risk of not having one’s advance directive honored constitutes a threat to a patient’s personal autonomy and liberty. Therefore, the personal identity debate is a threat to patients [1]. Is this argument better than the one above, which rested on the premise that the personal identity debate is metaphysical?

The problem with this other argument is that its conclusion does not follow from the premises, even if the premises are accepted as being correct. The main theme of Müller et al.’s article is that “the personal identity debate” is a threat to neurosurgical patients. And the various different perspectives discussed above are all introduced by Müller et al. as being part of the personal identity debate. But a lot of these different perspectives are not used – and probably couldn’t be used – to construct arguments either for or against the use of advance directives. So the argument Müller et al. offers cannot be used to argue that the whole debate constitutes a threat to patients’ personal autonomy and liberty by being a threat to the use of advance directives.

To be sure, there are arguments that have been presented in the literature whose intended conclusion is that sometimes advance directives should not be honored, where the arguments in questions have made use of ideas about personal identity over time. For example, Müller et al. refer to one such argument by Merkel [15]. So the premise that arguments using ideas about personal identity can be used in arguments against honoring some advance directives is true. And for the sake of discussion, we can also assume that such arguments can plausibly be said to pose a risk to patients’ personal autonomy and liberty. What these premises show, if correct, is that some views within the personal identity debate can be used in ways that may clash with some patients’ interests. But these premises do not support the much wider conclusion that Müller et al. draw, namely, that the personal identity debate is a general threat to patients’ personal autonomy and liberty.

Moreover, I argue in my article together with O'Neill that DBS-treatment can be seen – and is sometimes seen by patients – as having a positive effect on the patient’s self [9]. Some patients and their families – as well as their neurologists – judge that the patient’s true self, or a “better version” of the patient, is realized with the help of DBS. E.g. [16]. So as O'Neill and I were arguing in our article, the discussion of DBS within neuroethics should not be understood as only being about whether or not DBS poses a threat to patients’ self or identity. It is also about ways that DBS can benefit the self. Similarly, Baylis argues in her article in this journal that the idea that DBS poses a threat to patients’ identity is either “false, misleading, or trivially true.” For these reasons, Baylis argues, we should not worry too much about DBS’s posing threats to patients’ identity, other than in some extreme cases [6]. I fail to see how contributions to the personal identity debate such as my own just-mentioned article or Baylis’ article – both of which Müller et al. highlight as part of the “personal identity debate” – pose any threats to neurosurgical patients of the sorts that Müller et al. worry about. I conclude that the second key argument of Müller et al.’s paper rests on an unjustified overgeneralization.

## Bad Methodology?

Another strand of argument in Müller et al.’s article alleges that the personal identity debate rests on a faulty methodology. According to Müller et al., contributors to this debate cherry-pick statements made by patients and their families, while ignoring other statements – and they then use them as direct evidence for metaphysical theories they are supposedly trying to defend. This is a bad methodology, Müller et al. argue, because patients and their families’ statements are “vague”, “colloquial”, and “contradictory”. Hence they provide no evidence for the metaphysical theories that participants of the personal identity debate are trying to support using these statements. But do contributors to this debate really typically “use the interpretation of selected patient reports as evidence in support of a philosophically controversial theory”? [1].

In their article, Müller et al. do not produce any examples of any instances where contributors to the personal identity debate try to use the statements of patients or their families as direct evidence for any metaphysical theories. And I am not aware of any articles in this debate where patient-statements are presented as evidence for the theories of personal identity or the self that the articles discuss. Accordingly, it seems to me that Müller et al. are mistaken about what most contributors to this debate are trying to do.

True, most contributors to the debate bring up the sorts of the statements that patients, their families, and neurologists sometimes make about the self. The fact that these stakeholders make these kinds of statements when they are interviewed about or write about DBS is precisely the reason why there is a philosophical and neuroethical debate about the impact DBS can have on the self or personal identity. But bringing up these kinds of statements and organizing part of the discussion around those statements is not the same as using those statements as evidence in favor of one’s own favorite metaphysical theories. Nor, as I argued above, do all contributors of these debates seek to either defend or apply any metaphysical theories within the context of this debate.

It should also be acknowledged that some contributions to the personal identity debate about DBS focus primarily on what is perhaps a too narrow range of patient-outcomes, while failing to contextualize these within the broader range of patient-outcomes, many of which are highly positive. And so there is certainly room for methodological improvements in the personal identity debate. For example, it would be good to be more careful about situating the sorts of statements that some patients and their families make about DBS and the self within a much broader context.^2^ But that it would be good to contextualize these most-discussed patient-outcomes within a wider range of outcomes does not mean that most contributions to the personal identity debate rest on faulty methodology. Nor does it confirm that most writers in this debate proceed in the manner that Müller et al. describe them as proceeding. After all, focusing on a somewhat narrow sub-set of all patient-outcomes is not the same as using those patient-outcomes as evidence for contentious metaphysical theories.

Most participants of the debate are better understood as trying to interpret, understand, and empathize with the sorts of thoughts and concerns that some patients and their families have about the self and identity. They do this because they want to make sure that these thoughts and concerns are taken seriously, and not ignored or pushed aside within the neuroethical debate about DBS. One way of doing this is to relate patients and their families’ statements to theories of the self and identity that have been discussed in philosophy, or in other fields such as in social psychology or literary criticism. Many such theories attempt to understand and articulate common ideas and deep-seated intuitions that people have about the self and about their own and other people’s identity [9, 11, 12]. Surely there is nothing problematic, from a methodological point of view, with using the philosophical literature – along with other types of literature – to try to charitably interpret and take seriously the sorts of thoughts that patients, their families, and also neurologists have about DBS and its possible impact on the self.

It would strike me as more problematic, from an ethical point of view, to dismiss patients and their families’ statements as being “vague” and “contradictory” and, for these reasons, not worth discussing in a systematic way within the neuroethics of DBS. Thoughts and concerns about DBS’s actual or potential effects on the self and identity have come up many times in many real life cases. So, the neuroethics of DBS cannot ignore such thoughts and concerns. It should instead engage with them and take them seriously. And where patients and their families’ statements may appear less well-articulated and less thoroughly coherent than they could have been if they would have had time and opportunity to articulate these thoughts in a more systematic way, I see nothing wrong with trying to interpret them through the prism of widely discussed theories within philosophy. Doing so is a way of recognizing that patients and their families may have attitudes and thoughts that are important and widely shared, but that aren’t always easy to clearly articulate in a fully worked-out way.

## Conclusion

To be sure, it is very clear that Müller et al. have patients’ best interests at heart. And they are right that if some debate within neuroethics – or philosophy in general – would constitute a threat to patients and their best interests, then this would be a big problem with that debate. However, Müller et al.’s overall argument to the effect that the personal identity debate constitutes a threat to patients is problematic. For the reasons presented above, I think we can conclude that Müller et al. are not right to say that the personal identity debate is a threat to neurosurgical patients.

Let us end by briefly considering the alternative suggestion that Müller et al. make in their paper in light of what has been said above. They make what they call a “pragmatic” suggestion, according to which what is needed are:

(1) empirical research on personality changes arising from brain disorders and interventions, (2) comprehensive information about risks of personality changes, and (3) advance directives, particularly Ulysses contracts [1].

As before, these suggestions can certainly be seen as being made with the patients’ best interests in mind. However, it is a mistake to think that empirical research on personality changes can be done in a way that is wholly separate from any more philosophical questions about the self and identity. The ideas most people have about the self and identity are, as Levy puts it, typically part of “outlooks on human life” or “conceptions of how best to live”. Fully understanding the ethical importance of personality changes therefore requires that we bring in ethical theory as one part of the analysis of the possible effects of DBS.

Relatedly, in order for patients and their families to be able to understand and deliberate on the basis of “comprehensive information about risks of personality changes”, this information needs to be communicated to them. For this purpose, it is useful to have interpretations of what sorts of thoughts and concerns these key stakeholders are likely to have. With charitable interpretations of the sorts of concerns and thoughts many people have about the self and identity, practitioners can more easily communicate with patients about risks in a way that is responsive to widely shared concerns. To be sure, more empirical research is needed, just as Müller et al. suggest. However, empirical research alone is much less likely to deliver such interpretations than empirical research coupled with philosophical analysis is.

Lastly, it is surely right that respecting personal autonomy is very important within healthcare contexts and that one of the ways in which this can be done is for there to be advance directives and Ulysses contracts – at least within certain limits.^3^ However, a wider perspective would not only be concerned with respecting personal autonomy and liberty. It would also be concerned with respecting other values that many patients have, including values related to the self, identity, authenticity, etc. That’s another reason why the personal identity debate as a whole is not a threat to patients. Instead, it constitutes an attempt to take seriously values, thoughts, and concerns shared by many patients and their families.

## Footnotes

1 Understood in this way, metaphysics is largely independent of ethics. At least this must be how Müller et al. understand metaphysics since they say that the participants in the personal identity debate take metaphysical ideas, apply these to DBS-patients, and then, after that, make ethical arguments on this metaphysical basis [1].

2 Thanks to an anonymous reviewer for raising this point.

3 Müller et al. themselves also express the opinion that there can be certain limits to when it makes sense to honor advance directives.
